# Disordered eating and cardiometabolic risk factors in Chinese women: evidence from the China Health and Nutrition Survey

**DOI:** 10.1017/S0007114524001983

**Published:** 2024-09-14

**Authors:** Baiyu Qi, Gabrielle E. Cooper, Laura M. Thornton, Ruyue Zhang, Shuyang Yao, Annie Green Howard, Penny Gordon-Larsen, Shufa Du, Huijun Wang, Bing Zhang, Cynthia M. Bulik, Kari E. North, Melissa A. Munn-Chernoff

**Affiliations:** 1 Department of Epidemiology, Gillings School of Global Public Health, University of North Carolina at Chapel Hill, Chapel Hill, NC, USA; 2 Department of Psychiatry, University of North Carolina at Chapel Hill, Chapel Hill, NC, USA; 3 Department of Medical Epidemiology and Biostatistics, Karolinska Institutet, Stockholm, Sweden; 4 Department of Biostatistics, Gillings School of Global Public Health, University of North Carolina at Chapel Hill, Chapel Hill, NC, USA; 5 Carolina Population Center, University of North Carolina at Chapel Hill, Chapel Hill, NC, USA; 6 Department of Nutrition, Gillings School of Global Public Health, University of North Carolina at Chapel Hill, Chapel Hill, NC, USA; 7 National Institute for Nutrition and Health, Chinese Center for Diseases Control and Prevention, Beijing, PR China; 8 Department of Community, Family, and Addiction Sciences, Texas Tech University, Lubbock, TX, USA

**Keywords:** Epidemiology, Co-morbidity, Obesity, Global health

## Abstract

Disordered eating (DE) is associated with elevated cardiometabolic risk (CMR) factors, yet little is known about this association in non-Western countries. We examined the association between DE characteristics and CMR and tested the potential mediating role of BMI. This cross-sectional study included 2005 Chinese women (aged 18–50 years) from the 2015 China Health and Nutrition Survey. Loss of control, restraint, shape concern and weight concern were assessed using selected questions from the SCOFF questionnaire and the Eating Disorder Examination-Questionnaire. Eight CMR were measured by trained staff. Generalised linear models examined associations between DE characteristics with CMR accounting for dependencies between individuals in the same household. We tested whether BMI potentially mediated significant associations using structural equation modelling. Shape concern was associated with systolic blood pressure (*β* (95 % CI) 0·06 (0·01, 0·10)), diastolic blood pressure (DBP) (0·07 (95 % CI 0·03, 0·11)) and high-density lipoprotein (HDL)-cholesterol (–0·08 (95 % CI –0·12, −0·04)). Weight concern was associated with DBP (0·06 (95 % CI 0·02, 0·10)), triglyceride (0·06 (95 % CI 0·02, 0·10)) and HDL-cholesterol (–0·10 (95 % CI –0·14, −0·07)). Higher scores on DE characteristics were associated with higher BMI, and higher BMI was further associated with lower HDL-cholesterol and higher other CMR. In summary, we observed significant associations between shape and weight concerns with some CMR in Chinese women, and these associations were potentially partially mediated by BMI. Our findings suggest that prevention and intervention strategies focusing on addressing DE could potentially help reduce the burden of CMR in China, possibly through controlling BMI.

Cardiometabolic diseases, such as CVD and type 2 diabetes mellitus (T2DM), are major public health concerns in China. CVD is the leading cause of death in China^(1)^, and the prevalence of T2DM has increased from 1·3 % in 1980–1989 to 8·7 % in 2010–2014^(2)^. Approximately 60 % of Chinese women aged 18–40 years have at least one of the following cardiometabolic risk (CMR) factors: pre-diabetes/diabetes, hypertension, high low-density lipoprotein (LDL)-cholesterol, low high-density lipoprotein (HDL)-cholesterol, high triglyceride (TAG) and high total cholesterol^(3)^. Developing effective prevention strategies for CMR is a priority.

Eating disorders (EDs) and disordered eating (DE) could be important yet understudied risk factors for high CMR. According to the Diagnostic and Statistical Manual of Mental Disorders, Fifth Edition, EDs are psychiatric disorders characterised by a pathological disturbance in eating behaviours and attitudes and diagnosed according to specific criteria, including anorexia nervosa, bulimia nervosa, binge-eating disorder (BED), avoidant restrictive food intake disorder, other specified feeding and eating disorder, pica and rumination disorder^(4)^. On the other hand, DE includes a wide range of problems related to food, exercise and/or one’s body that do not warrant a diagnosis of a specific ED^(5)^. Previous studies have provided evidence on the associations between EDs and DE with cardiometabolic diseases and their risk factors in Western countries. For instance, bulimia nervosa and BED are both associated with a higher risk of T2DM, whereas anorexia nervosa is associated with a lower risk of T2DM, compared with controls^(6)^. Individuals with BED who have overweight or obesity are at a higher risk of reporting new diagnoses of dyslipidemia and hypertension than those without BED^(7)^. Bariatric surgery candidates with BED also have higher impaired fasting glucose levels and high TAG compared with those without BED^(8)^. As for DE characteristics, adults with higher scores on problematic relationships to eating and food (e.g. weight concerns and distress about overeating) were more likely to develop metabolic syndrome and T2DM compared with those who had lower scores^(9)^. In a large population-based cohort, the presence of objective binge eating was associated with higher fasting glucose and higher odds of hypertension, hypertriacylglycerolaemia, low HDL-cholesterol and insulin resistance compared with individuals without objective binge eating^(10)^. Furthermore, unhealthy weight control behaviours, including vomiting, skipping meals/fasting and laxative/diuretic use, have been associated with higher rates of incident diabetes among adults with overweight or obesity^(11)^. Lastly, in Latino adults, positive associations have emerged for emotional eating with T2DM diagnosis and hypertension, as well as for cognitive restraint with T2DM diagnosis and hyperlipidemia^(12)^.

BMI is potentially an important underlying factor mediating the associations between EDs or DE and CMR. Previous studies examined EDs or DE with CMR adjusted for BMI, yet results were mixed. For example, some associations were independent of BMI, including associations of BED with dyslipidemia and hypertension^(7)^, BED with fasting glucose levels and TAG^(8)^, and of binge-eating behaviours with hyperlipidemia^(11)^ among individuals with overweight or obesity. In contrast, some associations between EDs or DE with CMR were attenuated or became non-significant after adjusting for BMI, including associations of BED or problematic relationships with eating and food (e.g. weight concerns) and metabolic syndromes^(9,13)^, and objective binge eating with multiple CMR^(10)^. However, no formal test has been conducted to examine whether BMI mediates the association between DE and CMR. Understanding the precise role of BMI in these associations could provide a more comprehensive understanding of the association between DE and CMR.

Multiple gaps exist in the current literature. First, most previous studies were conducted in Western developed countries with European-ancestry populations. With an increasing prevalence of cardiometabolic diseases and risk factors over the past decades in China, investigating their association can aid public health workers in targeting at-risk populations and designing improved prevention and intervention strategies for both DE and cardiometabolic diseases. Second, most previous studies examined the association between ED diagnoses and CMR. Although published studies on DE have typically only focused on a few DE characteristics (e.g. problematic relationships to eating and food, objective binge eating, unhealthy weight control behaviours, emotional eating and cognitive restraint), individuals with DE characteristics had higher levels of CMR than those without such characteristics. Thus, DE characteristics alone are important risk factors of CMR. It is important to further explore the associations between a wider range of DE characteristics with CMR and determine specific associations of each characteristic with CMR. Third, studies should be designed to determine the extent to which any observed association between DE and CMR is potentially mediated by BMI. Accordingly, we investigate the association between DE behavioural and cognitive characteristics (i.e. loss of control eating (LOC), restraint, shape concern and weight concern) with eight CMR and explore the potential mediating role of BMI in a large population-based sample of women in mainland China. Findings from this study will provide more evidence on the associations between DE characteristics and CMR in Chinese women and elucidate mechanisms underlying these associations.

## Methods

### Design and participants

The China Health and Nutrition Survey (CHNS), a longitudinal open-cohort study, was initiated in 1989 with researchers’ desire to explore how economic and social change affected health behaviours in a large country^(14)^. The CHNS has been repeated every 2 to 4 years, resulting in eleven waves from 1989 to 2015. The study took place in fifteen provinces and municipal cities (i.e. Liaoning, Shandong, Henan, Jiangsu, Hubei, Hunan, Guizhou, Guangxi, Heilongjiang, Beijing, Chongqing, Shanghai, Shaanxi, Yunnan and Zhejiang) to represent areas with substantial difference in geography, economic development, public resources and health indicators^(15)^. Within each province or municipal city, a multistage, random cluster process was used to draw the study sample^(16)^. Data were collected at individual, household and community levels using various approaches such as questionnaires, face-to-face interview, phone interview for those who were not at home during data collection, asking community heads and health workers, and measurements by professional staff^(14)^. All participants provided signed informed consent prior to participation in the survey. CHNS was approved by Institutional Review Board at the University of North Carolina at Chapel Hill, Regional Ethical Review Board in Stockholm, and the Chinese Center for Disease Control and Prevention. The present study was approved by the Institutional Review Board at the University of North Carolina at Chapel Hill.

The current study used a cross-sectional design, and data were from the 2015 exam of the CHNS, which is the most recent database release for research. More than 7200 households composed of over 30 000 individuals from 216 communities were included in the 2015 wave. DE questions were measured directly as part of the CHNS individual survey for all female participants aged 12–50 years only; thus, it is not possible to include male participants or women with older ages. Female participants who completed questionnaires assessing DE characteristics and were measured for CMR during physical exams were included in this current study. We excluded participants who (1) were younger than 18 years, (2) had missing data on DE or CMR and (3) had implausible data on height or weight (e.g. one individual with height = 63 cm and weight = 55 kg).

### Measures

#### Disordered eating characteristics

As CHNS is a large population-based study assessing multiple domains of population health, the choices of screening tools were made to not only ensure information accuracy but also to reduce participants’ burden. Thus, the Chinese version of the SCOFF questionnaire^(17,18)^ was selected because of its brief scale that could capture several core features of EDs and DE. Seven questions from the Eating Disorder Examination-Questionnaire (EDE-Q v.6.0)^(19)^ were also asked to capture characteristics that were not covered by the SCOFF questionnaire.

Four DE characteristics were assessed: two behavioural characteristics (LOC and restraint) and two cognitive characteristics (shape concern and weight concern). LOC was assessed using one item from the SCOFF questionnaire – ‘Do you worry that you have lost control over how much you eat?’ Response options were ‘Yes’ or ‘No’. Restraint, shape concern and weight concern during the past 28 d were assessed using seven items from the EDE-Q v.6.0, including five items from the full restraint subscale, one item – ‘Have you felt fat?’ – from the shape concern subscale and one item – ‘Have you had a strong desire to lose weight?’ – from the weight concern subscale. Each item was answered on a six-point scale ranging from ‘*No*’ to ‘*Daily*’. Restraint scores were calculated by taking the mean of the five items from the restraint subscale. Each characteristic score ranged from 0 to 6, with a higher score indicating greater psychopathology. In our sample, the restraint subscale had a standardised Cronbach’s α of 0·80.

#### Cardiometabolic risk factors

Eight continuous CMR were included in our study. Systolic blood pressure (SBP) and diastolic blood pressure (DBP) were calculated as means of three measurements by trained examiners. Fasting blood was collected and immediately tested for serum glucose. The remaining blood samples were then prepared for further testing in a national central laboratory in Beijing (medical laboratory accreditation certificate ISO 15189:2007). HbA1c was measured using an automated glycohemoglobin analyser with a HPLC system (model HLC-723 G7; Tosoh Corp). Fasting lipids (i.e. HDL-cholesterol, LDL-cholesterol, total cholesterol and TAG) were measured using the glycerol phosphate oxidase method. All measurements and tests were conducted using standard protocols by trained staff. The detailed data collection protocol has been previously described^(3,14)^.

Seven binary CMR, based on these eight continuous CMR, were generated. Hypertension was defined as mean SBP ≥ 140 mmHg or mean DBP ≥ 90 mmHg, according to the WHO^(20)^, or self-reported use of antihypertension medications. T2DM was defined as HbA1c ≥ 6·5 % or fasting glucose ≥ 126 mg/dl, according to the American Diabetes Association^(21)^, or self-reported use of T2DM medications or insulin. Pre-diabetes was defined as 5·7 % ≤ HbA1c < 6·5 % or 100 mg/dl ≤ fasting glucose < 126 mg/dl or self-reported use of medications for pre-diabetes. Low HDL-cholesterol was defined as HDL-cholesterol < 50 mg/dl. High LDL-cholesterol, high total cholesterol and high TAG were defined as LDL-cholesterol ≥ 130 mg/dl, total cholesterol ≥ 200 mg/dl and TAG ≥ 150 mg/dl, respectively^(22)^. We summarised descriptive statistics for binary CMR as they provide more clinical significance but did not include them in the subsequent analyses because (1) it is more informative to examine the entire distribution of each factor as the thresholds for defining binary CMR are arbitrary and (2) the statistical power to detect effects is higher when considering a continuous CMR, compared with the power of analysing CMR as a binary trait.

#### BMI and sociodemographic characteristics

Height and weight were measured by trained staff during physical exams. Height was measured without shoes to the nearest 0·1 cm using portable stadiometers. Weight was measured in light clothing to the nearest 0·1 kg using calibrated beam scales. BMI (kg/m^2^) was calculated as weight in kilograms divided by height in metres squared.

Sociodemographic variables including age (calculated as year of survey minus birth year), geographic region (north or south), setting (urban or rural) and education level (categorised as none or primary education, middle education, technical or vocational degree, and university degree or higher) were asked in the CHNS individual survey. Per capital household income (in Chinese Yuan) was asked in the CHNS household survey.

### Statistical analysis

Descriptive and multiple regression analyses were conducted in SAS 9.4 (SAS Institute, 2013). Descriptive statistics of demographic characteristics, DE characteristics, and continuous and binary CMR were summarised for the whole sample.

To examine the association between each DE and each continuous CMR, we used generalised linear models modeling each pairwise combination of CMR outcome and DE characteristic. To maintain a parsimonious regression model and avoid over-adjustment, we exclusively accounted for age and education level as confounders, as they were the only covariates associated with both DE characteristics and CMR in our sample. All models accounted for dependencies between individuals in the same household by applying generalised estimating equations which used the Liang–Zeger sandwich estimator^(23)^. Standardised regression coefficients with 95 % CI are presented. For a total of thirty-two regression models, multiple comparisons were adjusted using false discovery rate correction^(24)^. Associations with a false discovery rate-adjusted *P*-value (q-value) < 0·05 were considered statistically significant.

**Fig. 1.**
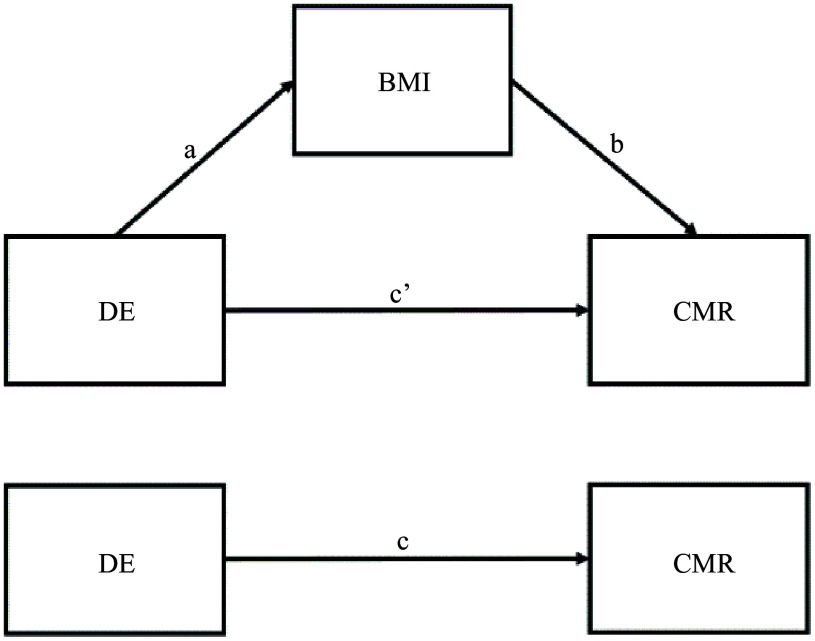
A mediation model with BMI mediating the association between a disordered eating (DE) characteristic with a cardiometabolic risk (CMR). a = potential effect of DE on BMI; b = potential effect of BMI on CMR; c’ = potential direct effect of DE on CMR; a × b = potential indirect (mediating) effect of DE on CMR through BMI; a × b + c’ = c (potential total effect of DE on CMR).

For significant associations between DE characteristics and CMR, we then conducted mediation analysis to test whether BMI potentially mediated the associations by fitting structural equation models using the ‘lavaan’ package^(25)^ in R^(26)^. A mediation model (Fig. 1) simultaneously fits a set of regressions between DE with BMI (path a), BMI with CMR (path b) and DE with CMR while holding BMI constant (path c’; potential direct effect). The potential indirect (mediating) effect (a × b) measures the associations of DE and CMR resulting from the associations of DE with BMI, which in turn is associated with the CMR. The sum of potential direct and indirect effects is equal to the potential total effect (c) of DE on CMR. Age and education level were included as covariates in all pathways described in the mediation models. Potential standardised average total effects, average direct effects and average indirect effects of DE characteristics on CMRs were calculated. The 95 % CI for potential effects were calculated by running 1000 simulations using the non-parametric bootstrapping method to correct for non-normality and address power limitations^(27)^. Results with *P*-values < 0·05 were considered statistically significant. Notably, due to the cross-sectional design, estimated standardised coefficients reflect correlation coefficients rather than causal effects.

To examine potential effect modification by age, we conducted additional sensitivity analysis by replicating all analyses separately in two age groups: those aged 18–40 years and those older than 40 years. We selected 40 as the cut-off value for two primary reasons. First, the median age of our sample was 40 years; thus, it could result in two sub-samples with comparable sample size and sufficient statistical power for each sample. Second, according to the National Institute for Health and Care Excellence Clinical Guideline on Cardiovascular Diseases, CMR assessment and reduction, including lipid medication, is recommended for those aged 40 years or older^(28)^.

## Results

A total of 4220 female participants completed questionnaires assessing DE characteristics. Of these participants, 2434 were measured for CMR during physical exams. After excluding participants who were younger than 18 years (*n* 146), had missing data on DE (*n* 15) or CMR (*n* 251) or had implausible data on height or weight (*n* 17), 2005 participants were included in the analysis. Table 1 presents participant characteristics for the full sample. Missing values only existed for per capita household income (n_miss_ = 51) and education (n_miss_ = 126). Participants had a mean age of 38 years (sd = 8) and a mean BMI of 24 kg/m^2^ (sd = 4). Compared with demographic characteristics of Chinese women in 2015 according to the China Statistical Yearbook^(29)^, our sample had a higher proportion of women who were located in the South (62 % *v*. 47 %) and who had a university degree or higher (20 % *v*. 6 %). Our sample also had a higher mean per capita household income (23 521 Chinese Yuan *v*. 21 966 Chinese Yuan) and lower proportion of those living in urban areas (34 % *v*. 56 %) compared with the Chinese population in 2015^(29)^. A total of 13 % (*n* 258) of the participants had hypertension, 4 % (*n* 74) had T2DM, 23 % (*n* 461) had high total cholesterol, 50 % (*n* 1012) had low HDL-cholesterol, 22 % (*n* 451) had high LDL-cholesterol and 15 % (*n* 298) had high TAG.

**Table 1.** Characteristics of female adults in the 2015 wave of the Chinese Health and Nutrition Survey (*n* 2005) (Mean values and standard deviations; numbers and percentages)

Demographic characteristics	Mean	sd
Age (year)	38·38	7·97
BMI (kg/m^2^)	23·51	3·76
Per capita household income (Chinese Yuan)*	23 521·27	41 480·83
Demographic characteristics	*n*	%
Geographic region		
North	758	37·81
South	1247	62·19
Setting		
Rural	1324	66·03
Urban	681	33·97
Education^†^		
None or primary education	274	13·67
Middle education	1007	50·22
Technical or vocational degree	193	9·63
University degree or higher	405	20·20
DE characteristics (cognitive)	Mean	sd
Shape concern	0·39	1·33
Weight concern	0·27	1·10
DE characteristics (behavioural)	Mean	sd
Restraint	0·09	0·41
DE characteristics (behavioural)	*n*	%
Loss of control eating	105	5·24

Continuous cardiometabolic risks	Mean	sd
SBP (mm Hg)	117·26	15·35
DBP (mm Hg)	76·84	10·10
HbA1c (%)	5·40	0·61
Glucose (mg/dl)	92·75	18·37
Total cholesterol (mg/dl)	178·01	38·18
HDL-cholesterol (mg/dl)	50·67	11·58
LDL-cholesterol (mg/dl)	109·28	31·24
TAG (mg/dl)	98·88	60·37
Binary cardiometabolic risks	*n*	%
Hypertension	258	12·87
T2DM	74	3·69
High total cholesterol	461	22·99
Low HDL-cholesterol	1012	50·47
High LDL-cholesterol	451	22·49
High TAG	298	14·86

DE, disordered eating; SBP, systolic blood pressure; DBP, diastolic blood pressure; HDL, high-density lipoprotein; LDL, low-density lipoprotein; TAG, triglyceride; T2DM, type 2 diabetes mellitus.

*Fifty-one participants had missing data for per capita household income.

^†^126 participants had missing data for education.

Table 2 shows the standardised results from generalised estimating equations estimating the association between DE characteristics with continuous CMR, adjusted for age and education level and accounted for dependencies between individuals in the same household. Only continuous CMR were included to capture the entire distribution of each CMR and to ensure sufficient statistical power. Six statistically significant (*q* < 0·05) associations were noted: one standard *increase* in shape concern score was associated with a 0·06 (95 % CI 0·01, 0·10) mmHg standard *increase* in SBP, 0·07 (95 % CI 0·03, 0·11) mmHg standard *increase* in DBP and 0·08 (95 % CI 0·04, 0·12) mg/dl standard *decrease* in HDL-cholesterol; one standard *increase* in weight concern score was associated with a 0·06 (95 % CI 0·02, 0·10) mmHg standard *increase* in DBP, 0·06 (95 % CI 0·02, 0·10) mg/dl standard *increase* in TAG and 0·10 (95 % CI 0·07, 0·14) mg/dl standard *decrease* in HDL-cholesterol.

**Table 2.** Standardised regression coefficients with 95 % CI from generalised estimating equations evaluating the effect of disordered eating characteristics on cardiometabolic risks (Standardised regression coefficients and 95 % confidence intervals)

	LOC		Restraint		Shape concern		Weight concern	
	Standardised regression coefficients	95 % CI	Standardised regression coefficients	95 % CI	Standardised regression coefficients	95 % CI	Standardised regression coefficients	95 % CI
SBP (mm Hg)	–0·03	–0·20, 0·15	0·01	–0·03, 0·05	0·06	0·01, 0·10*	0·05	0·01, 0·08
DBP (mm Hg)	0·01	–0·17, 0·19	0·02	–0·02, 0·06	0·07	0·03, 0·11*	0·06	0·02, 0·10*
HbA1c (%)	0·02	–0·15, 0·19	0·05	–0·04, 0·14	0·04	–0·01, 0·08	0·06	0·01, 0·12
Glucose (mg/dl)	0·02	–0·13, 0·18	0·04	–0·03, 0·12	0·02	–0·02, 0·06	0·06	0·00, 0·12
Total cholesterol (mg/dl)	–0·10	–0·27, 0·08	0·00	–0·05, 0·05	0·00	–0·03, 0·04	0·00	–0·04, 0·04
HDL-cholesterol (mg/dl)	–0·20	–0·40, 0·01	–0·04	–0·09, 0·01	–0·08	–0·12, −0·04*	–0·10	–0·14, −0·07*
LDL-cholesterol (mg/dl)	–0·03	–0·22, 0·16	–0·01	–0·05, 0·04	0·03	–0·01, 0·06	0·02··	–0·02, 0·06
TAG (mg/dl)	0·00	–0·19, 0·19	0·03	–0·02, 0·08	0·04	0·00, 0·08	0·06	0·02, 0·10*

LOC, loss of control eating; SBP, systolic blood pressure; DBP, diastolic blood pressure; HDL, high-density lipoprotein; LDL, low-density lipoprotein; TAG, triglyceride.

Models adjusted for age and education level and accounted for dependencies between individuals in the same household.

*q < 0·05.

For the six significant associations between DE characteristics and CMR, we fitted mediation models to test the potential mediating role of BMI (Table 3). For the association between weight concern and HDL-cholesterol, both potential direct and indirect effects were significant (*P* < 0·05), indicating that BMI only potentially partially mediated this association. More specifically, one standardised increase in weight concern was associated with a 0·65 (95 % CI 0·48, 0·83) standardised increase in BMI, and this subsequent 0·65 standardised increase in BMI was associated with a 0·04 (95 % CI 0·03, 0·06) decrease in HDL-cholesterol, given that each one standardised increase in BMI was associated with a 0·07 (95 % CI 0·06, 0·08) standardised decrease in HDL-cholesterol. The potential indirect effects of weight concern on HDL-cholesterol through BMI accounted for 41 % (95 % CI 29 %, 71 %) of the potential total effect of weight concern on HDL-cholesterol. For all other associations, the potential indirect effects of DE characteristics on CMR through BMI were significant, but the potential direct effects of DE characteristics on CMR were non-significant. The proportion of potential total effects of DE characteristics on CMR accounted for by potential indirect effects through BMI ranged from 60 % to 87 %, suggesting that BMI plays a large role in mediating these associations. For example, for the association between shape concern and SBP that was primarily comprised of the indirect effect through BMI, one standardised increase in shape concern was associated with a 0·71 (95 % CI 0·54, 0·89) standardised increase in BMI, and this subsequent 0·71 standardised increase in BMI was associated with a 0·05 (95 % CI 0·04, 0·07) increase in SBP, given that each one standardised increase in BMI was associated with a 0·07 (95 % CI 0·06, 0·09) standardised increase in SBP.

**Table 3.** Potential standardised average total, direct and indirect (mediating) effects with bootstrapped 95 % CI in mediation models

Association	Potential standardised total effect (c)	95 % CI	Standardised a	95 % CI	Standardised b	95 % CI	Potential standardised direct effect (c’)	95 % CI	Potential standardised indirect effect (a × b)	95 % CI	(a × b)/c^†^	95 % CI
Shape concern – SBP	0·06	0·01, 0·10*	0·71	0·54, 0·89*	0·07	0·06, 0·09*	0·01	–0·03, 0·05	0·05	0·04, 0·07*	84 %	60 %, 494 %
Shape concern – DBP	0·07	0·03, 0·11*	0·71	0·54, 0·89*	0·07	0·06, 0·09*	0·02	–0·02, 0·06	0·05	0·04, 0·07*	77 %	54 %, 231 %
Shape concern – HDL-cholesterol	–0·08	–0·12, −0·04*	0·71	0·54, 0·89*	–0·07	–0·08, −0·06*	–0·03	–0·07, 0·01	–0·05	–0·06, −0·03*	60 %	44 %, 580 %
Weight concern – DBP	0·06	0·02, 0·10*	0·65	0·48, 0·83*	0·07	0·06, 0·09*	0·01	–0·03, 0·05	0·05	0·03, 0·06*	82 %	54 %, 337 %
Weight concern – HDL-cholesterol	–0·10	–0·14, −0·07*	0·65	0·48, 0·83*	–0·07	–0·08, −0·06*	–0·06	–0·10, −0·03*	–0·04	–0·06, −0·03*	41 %	29 %, 71 %
Weight concern – TAG	0·06	0·02, 0·10*	0·65	0·48, 0·83*	0·06	0·04, 0·07*	0·02	–0·02, 0·06	0·04	0·02, 0·05*	65 %	44 %, 1631 %

a, potential effect of disordered eating (DE) on body mass index (BMI); b, potential effect of BMI on cardiometabolic risk (CMR); c’, potential direct effect of DE on CMR; c, potential total effect of DE on CMR; a × b, potential indirect (mediating) effect of DE on CMR through BMI; SBP, systolic blood pressure; DBP, diastolic blood pressure; HDL, high-density lipoprotein; TAG, triglyceride.

All models were adjusted for age and education level.

*P < 0·05.

^**†**^Proportion of total effects of DE on CMR (c) accounted by indirect effects through BMI (a × b).

Results from the sensitivity analysis suggested that the association between DE with CMR and the potential mediating role of BMI were homogeneous across age groups. Compared with participants aged 18–40 years (*n* 1034), those who were older than 40 years (*n* 971) had significantly higher BMI and increased levels of CMR, as well as decreased levels of educational attainment and lower scores on weight concern and LOC (online Supplementary Table 1). Both age groups displayed similar patterns of association between DE and CMR: weight concern was negatively associated with HDL-cholesterol in both age groups, and shape concern was positively associated with DBP in the older age group (*q* < 0·05; online Supplementary Table 2). In the mediation analysis (online Supplementary Table 3), BMI partially (46 % (95 % CI 31 %, 94 %)) mediated the association between weight concern with HDL-cholesterol in the younger group, with a standardised potential direct effect of –0·06 (95 % CI –0·11, –0·01). BMI fully mediated both associations for the older group, although the standardised potential direct effect of weight concern on HDL-cholesterol suggested borderline significance (–0·06 (95 % CI –0·11, 0·00)).

## Discussion

Our study examined the association between four DE characteristics with eight CMR and investigated the potential mediating role of BMI on these associations in a sample of Chinese women. Our findings suggest that Chinese women with higher shape or weight concern scores are more likely to have higher CMR compared with those with lower shape and weight concern scores, and BMI at least partially mediated these associations. These findings were homogeneous across age groups.

Shape and weight concerns were associated with multiple CMR, such as an elevated level of SBP, DBP, TAG and a lower level of HDL-cholesterol, consistent with previous studies^(30)^. Previous studies have also reported significant associations between DE and T2DM^(9,11,12)^, yet we did not find significant associations between DE with either HbA1c or glucose, possibly due to the low prevalence (3·69 %) of T2DM in our sample. With the exception of the association between weight concern and HDL-cholesterol, all other significant associations could likely be driven by BMI. Interestingly, previous studies have reported significant associations between EDs and DE with CMR after adjusting for BMI, such as positive associations between BED diagnosis or binge-eating behaviours with impaired glucose level and TAG^(8)^, risk of metabolic syndrome components^(7)^ and incident diabetes^(11)^, suggesting that BMI does not completely account for these associations. In our study, BMI at least partially mediated significant associations between shape and weight concerns with CMR. Thus, both the previous literature and our mediation results suggest that the DE–CMR associations are beyond the simple effect of BMI. For example, binge-eating behaviour may directly cause immediate metabolic consequences in healthy adults, such as decreased insulin sensitivity^(31)^, which could also explain associations with the CMR observed herein.

Only shape and weight concerns were significantly associated with CMR in our study. Both characteristics were significantly associated with DBP and HDL-cholesterol. Although shape concern was separately associated with SBP and weight concern was separately associated with TAG, the differences in statistical significance might be due to lack of statistical power. The similar pattern of associations between shape and weight concerns with CMR could be explained by the related nature between two traits (i.e. both traits reflect body dissatisfaction), as well as their moderate to high correlation (*r* = 0·67) in our sample. Shape and weight concerns are cognitive DE characteristics and can be present in individuals with high, normal or low BMI, manifesting as a preoccupation with shape and weight on a spectrum of normative discontent full-threshold EDs^(32,33)^. In our sample, the mean BMI was 23·51 kg/m^2^ and shape and weight concerns were positively correlated with BMI, suggesting that the scales were tapping into appearance concerns related to living in higher weight bodies. Further, the high BMI partially to fully explained the positive associations between shape and weight concerns with CMR. Interestingly, restraint was not significantly associated with any CMR in our sample, which contrasts findings from a previous study where cognitive restraint was associated with elevated odds of T2DM and hyperlipidemia^(12)^. One possible reason of the inconsistency between our results and previous findings is that restraint was only endorsed by very few participants our sample: the mean score on restraint was 0·09 (se = 0·41), which was significantly lower than the scores on shape (mean = 0·39, se = 1·33) and weight concern (Mean = 0·27, se = 1·10). In Lopez-Cepero *et al.* ^(12)^, 34 % of participants reported a high level of cognitive restraint, defined as having an above-median score on the cognitive restraint subscale of the Three-Factor Eating Questionnaire. Additionally, restraint was not significantly correlated with BMI in our sample, whereas high cognitive restraint was positively associated with odds of obesity and central obesity (defined as having a waist circumference > 35 in for women and > 40 in for men) in Lopez-Cepero *et al.* ^(12)^. However, Lopez-Cepero *et al.* ^(12)^ did not adjust for BMI in their models; thus, whether the significant association between cognitive restraint with CMR was due to high BMI remains unknown. Similarly, LOC was not correlated with BMI nor associated with any CMR in our sample. Although no previous study has examined the association between LOC with CMR, multiple studies reported a positive association between binge eating or BED with CMR independent of BMI^(6–8,10)^. As LOC is a core feature of binge eating, one hypothesis is that the association between binge eating or BED with CMR was either due to other features of the behaviour or disorder, such as ‘eating a large amount of food’ or ‘eating much more rapidly than normal’. More research is needed to clarify the roles of different DE characteristics on the risks of CMR.

Weight concern was negatively associated with HDL-cholesterol, and this association was only partially mediated by BMI, indicating that BMI is not the only mechanism contributing to the association between weight concern with HDL-cholesterol. This suggests that a high score on weight concern in our sample might also be driven by other related risk factors, such as certain behavioural factors that further contribute to HDL-cholesterol. One possible risk factor is physical activity, which is associated with low levels of HDL-cholesterol^(34)^. Although the association between weight concern with levels of physical activity remains unknown, some studies in individuals with BED have shown that those with a negative body attitude had lower levels of physical activity, compared with those with a less negative body attitude^(35)^. Thus, individuals who are concerned about their weight may also have low levels of physical activity, which may further impact HDL-cholesterol levels. Similarly, multiple dietary factors have been shown to affect HDL-cholesterol concentrations: for example, the substitution of saturated, monounsaturated or polyunsaturated fat for carbohydrates elevated HDL-cholesterol^(36)^. In contrast, added sugar consumption was negatively associated with HDL-cholesterol levels^(36)^. Few studies have examined the association of weight concern or other DE characteristics with dietary patterns. In one study, individuals with an ED reported a lower intake of dietary fat, and those with bulimia nervosa also reported a higher intake of refined sugar, when compared with those without an ED^(37)^. These findings suggest that dietary factors might also play a role in the association between weight concern with HDL-cholesterol. Another factor that potentially contributes to the association between weight concern and low HDL-cholesterol is smoking: a population-based study suggested that individuals who smoked daily had higher levels of weight concerns compared with those who recently quit smoking and former and occasional smokers^(38)^. Given that smoking is a well-established risk factor of low HDL-cholesterol independent of BMI^(39)^, it could be another pathway contributing to the association between weight concern and HDL-cholesterol. Indeed, the prevalence of low HDL-cholesterol (< 50 mg/dl) in our sample was 50·47 % – a much higher percentage than other CMR – suggesting that low HDL-cholesterol is common despite the relatively young age of our sample. Higher HDL-cholesterol levels are associated with protection against CHD^(40)^. Thus, future studies should further explore the role of these modifiable factors in the association between DE and HDL-cholesterol to better design targeted prevention and intervention.

To our knowledge, this is the first study to examine the associations between DE and CMR in China. We used a large sample of Chinese women and examined a wide range of DE characteristics and CMR. Nevertheless, our study had several limitations. First, findings may not be generalisable to all Chinese women due to the differences in demographic characteristics between our sample and the general Chinese population. For example, our sample had a higher proportion of women with at least a college degree than other women in China. As DE has been found to be positively associated with education level in China^(41)^, the prevalence of DE characteristics may be higher in our sample than other Chinese women. Our sample also only included women between 18 and 50 years old with a mean age of 38 years, yet most CMR, such as dyslipidemia and hypertension, are more prevalent in individuals aged 50 years or higher^(42,43)^. Additionally, DE was only assessed in women due to limited funding. Given the sex difference in DE and EDs^(44)^, as well as cardiometabolic disorders^(45)^, future studies should further examine sex differences in the association between DE and CMR in the general population. Thus, our findings may only be generalised to Chinese women aged 18–50 years with a high education level. Second, LOC, shape concern and weight concern were assessed by a single question due to space limitations in the large survey, which should be considered when interpreting results. We were also not able to assess ED pathology using the full EDE-Q or ask about history of ED diagnosis due to limited space in the survey. Third, repeated measurements of CMR could reduce random errors, which was lacking in our study. Most CMR (e.g. HbA1c and fasting glucose) were only measured once due to the large scale of the survey and limited time allocated for each exam. However, the moderate to large sample size of our study may still control random errors to some extent. Lastly, given the cross-sectional design of this study, we did not aim to infer causality or directionality of the associations among DE, BMI and CMR. For example, our estimates could be subject to unmeasured confounding and/or the association between DE and CMR in our sample could be bidirectional. Rather, we aimed to examine the magnitude of variation in CMR due to DE that would remain if an intermediate risk factor was changed. A longitudinal study is necessary to further understand the temporal sequence between DE and CMR.

Our study provided evidence of the association between shape and weight concerns with CMR in Chinese women, and these associations were at least partially mediated by BMI. Weight concern was to some degree associated with low HDL-cholesterol independent of BMI. These findings suggest that screening, prevention and intervention focusing on DE characteristics could potentially reduce the growing burden of CMR in Chinese women by controlling BMI. For example, for women with higher BMI, healthcare providers may consider screening for DE characteristics when clinically evaluating BMI and other CMR. DE characteristics are modifiable and may represent an important intervention target for reducing CMR factors in women. Strategies for prevention and intervention for reducing DE among Chinese women in the context of CMR management are also needed. Future studies should replicate these results using more validated scales and, potentially, in older female adults and in males. A longitudinal design will assist in establishing temporal sequence of these associations.

## Supporting information

Qi et al. supplementary material Qi et al. supplementary material

## Supplementary material

For supplementary material/s referred to in this article, please visit https://doi.org/10.1017/S0007114524001983

## References

[1] Ma LY , Chen WW , Gao RL , et al. (2020) China cardiovascular diseases report 2018: an updated summary. J Geriatr Cardiol 17, 1–8. 32133031 10.11909/j.issn.1671-5411.2020.01.001 PMC7008101

[2] Yuan H , Li X , Wan G , et al. (2018) Type 2 diabetes epidemic in East Asia: a 35-year systematic trend analysis. Oncotarget 9, 6718–6727. 29467922 10.18632/oncotarget.22961 PMC5805508

[3] Yan S , Li J , Li S , et al. (2012) The expanding burden of cardiometabolic risk in China: the China Health and Nutrition Survey. Obes Rev 13, 810–821. 22738663 10.1111/j.1467-789X.2012.01016.x PMC3429648

[4] Regier DA , Kuhl EA & Kupfer DJ (2013) The DSM-5: classification and criteria changes. World Psychiatry 12, 92–98. 23737408 10.1002/wps.20050 PMC3683251

[5] Pereira RF & Alvarenga M (2007) Disordered eating: identifying, treating, preventing, and differentiating it from eating disorders. Diabetes Spectr 20, 141–148.

[6] Nieto-Martinez R , Gonzalez-Rivas JP , Medina-Inojosa JR , et al. (2017) Are eating disorders risk factors for type 2 diabetes? A systematic review and meta-analysis. Curr Diab Rep 17, 138. 29168047 10.1007/s11892-017-0949-1

[7] Hudson JI , Lalonde JK , Coit CE , et al. (2010) Longitudinal study of the diagnosis of components of the metabolic syndrome in individuals with binge-eating disorder. Am J Clin Nutr 91, 1568–1573. 20427731 10.3945/ajcn.2010.29203 PMC2869508

[8] Mitchell JE , King WC , Pories W , et al. (2015) Binge eating disorder and medical comorbidities in bariatric surgery candidates. Int J Eat Disord 48, 471–476. 25778499 10.1002/eat.22389 PMC4980070

[9] Yoon C , Jacobs DR Jr , Duprez DA , et al. (2019) Problematic eating behaviors and attitudes predict long-term incident metabolic syndrome and diabetes: the Coronary Artery Risk Development in Young Adults Study. Int J Eat Disord 52, 304–308. 30636022 10.1002/eat.23020 PMC6408221

[10] Abraham TM , Massaro JM , Hoffmann U , et al. (2014) Metabolic characterization of adults with binge eating in the general population: the Framingham Heart Study. Obesity (Silver Spring) 22, 2441–2449. 25136837 10.1002/oby.20867 PMC4224974

[11] Nagata JM , Garber AK , Tabler J , et al. (2018) Disordered eating behaviors and cardiometabolic risk among young adults with overweight or obesity. Int J Eat Disord 51, 931–941. 30030944 10.1002/eat.22927 PMC6230303

[12] Lopez-Cepero A , Frisard CF , Lemon SC , et al. (2018) Association of dysfunctional eating patterns and metabolic risk factors for cardiovascular disease among Latinos. J Acad Nutr Diet 118, 849–856. 28774505 10.1016/j.jand.2017.06.007

[13] Leone A , Bedogni G , Ponissi V , et al. (2016) Contribution of binge eating behaviour to cardiometabolic risk factors in subjects starting a weight loss or maintenance programme. Br J Nutr 116, 1984–1992. 27974060 10.1017/S0007114516004141

[14] Popkin BM , Du S , Zhai F , et al. (2010) Cohort profile: the China Health and Nutrition Survey--monitoring and understanding socio-economic and health change in China, 1989–2011. Int J Epidemiol 39, 1435–1440. 19887509 10.1093/ije/dyp322 PMC2992625

[15] Zhang B , Zhai FY , Du SF , et al. (2014) The China Health and Nutrition Survey, 1989–2011. Obes Rev 15, 2–7. 10.1111/obr.12119 PMC3869031 24341753

[16] Popkin BM (1998) The nutrition transition and its health implications in lower-income countries. Public Health Nutr 1, 5–21. 10555527 10.1079/phn19980004

[17] Morgan JF , Reid F & Lacey JH (1999) The SCOFF questionnaire: assessment of a new screening tool for eating disorders. BMJ 319, 1467–1468. 10582927 10.1136/bmj.319.7223.1467 PMC28290

[18] Leung SF , Lee KL , Lee SM , et al. (2009) Psychometric properties of the SCOFF questionnaire (Chinese version) for screening eating disorders in Hong Kong secondary school students: a cross-sectional study. Int J Nurs Stud 46, 239–247. 18945428 10.1016/j.ijnurstu.2008.09.004

[19] Fairburn CG & Beglin SJ (2008) Eating disorder examination questionnaire. Cognit Behav Ther Eat Disord 309, 313.

[20] Whitworth JA , World Health Organization & ISoHWG (2003) 2003 World Health Organization (WHO)/International Society of Hypertension (ISH) statement on management of hypertension. J Hypertens 21, 1983–1992. 14597836 10.1097/00004872-200311000-00002

[21] Care D (2017) Classification and diagnosis of diabetes. Diabetes Care 40, S11–S24. 27979889 10.2337/dc17-S005

[22] Panel III AT (2001) Guidelines At-a-Glance Quick Desk Reference. National Cholesterol Education Program (NCEP). https://www.nhlbi.nih.gov/files/docs/guidelines/atglance.pdf (accessed 10 May 2021).

[23] Liang K-Y & Zeger SL (1986) Longitudinal data analysis using generalized linear models. Biometrika 73, 13–22.

[24] Benjamini Y & Hochberg Y (1995) Controlling the false discovery rate: a practical and powerful approach to multiple testing. J R Stat Soc Ser B Stat Methodol 57, 289–300.

[25] Rosseel Y (2012) lavaan: an R package for structural equation modeling. J Stat Softw 48, 1–36.

[26] R Core Team (2013) R: A Language and Environment for Statistical Computing. R Foundation for Statistical Computing. Vienna, Austria: R Core Team. http://www.R-project.org/

[27] Preacher KJ & Hayes AF (2008) Assessing mediation in communication research. In The Sage Sourcebook of Advanced Data Analysis Methods for Communication, pp. 13–54 [ AF Hayes , MD Slater and LB Snyder , editors]. Thousand Oaks, CA: Sage Publications.

[28] Duerden M , O’Flynn N & Qureshi N (2015) Cardiovascular risk assessment and lipid modification: NICE guideline. Br J Gen Pract 65, 378–380. 26120133 10.3399/bjgp15X685933 PMC4484941

[29] China NBoSo (2016) China Statistical Yearbook 2016. https://www.stats.gov.cn/sj/ndsj/2016/indexeh.htm (accessed September 2023).

[30] Bell JA , Carslake D , O’Keeffe LM , et al. (2018) Associations of body mass and fat indexes with cardiometabolic traits. J Am Coll Cardiol 72, 3142–3154. 30545453 10.1016/j.jacc.2018.09.066 PMC6290112

[31] Parry SA , Woods RM , Hodson L , et al. (2017) A single day of excessive dietary fat intake reduces whole-body insulin sensitivity: the metabolic consequence of binge eating. Nutrients 9, 818. 28758920 10.3390/nu9080818 PMC5579612

[32] Rodin J , Silberstein L & Striegel-Moore R (1984) Women and weight: a normative discontent. Nebr Symp Motiv 32, 267–307. 6398857

[33] Fairburn CG & Beglin SJ (1994) Assessment of eating disorders: interview or self-report questionnaire? Int J Eat Disord 16, 363–370. 7866415

[34] Rosenson RS (2005) Low HDL-C: a secondary target of dyslipidemia therapy. Am J Med 118, 1067–1077. 16194634 10.1016/j.amjmed.2004.12.021

[35] Vancampfort D , Vanderlinden J , Stubbs B , et al. (2014) Physical activity correlates in persons with binge eating disorder: a systematic review. Eur Eat Disord Rev 22, 1–8. 24014460 10.1002/erv.2255

[36] Siri-Tarino PW (2011) Effects of diet on high-density lipoprotein cholesterol. Curr Atheroscler Rep 13, 453–460. 21901431 10.1007/s11883-011-0207-y

[37] Allen KL , Mori TA , Beilin L , et al. (2013) Dietary intake in population-based adolescents: support for a relationship between eating disorder symptoms, low fatty acid intake and depressive symptoms. J Hum Nutr Diet 26, 459–469. 23216519 10.1111/jhn.12024

[38] Luostarinen M , Tuovinen EL , Saarni SE , et al. (2013) Weight concerns among Finnish ever-smokers: a population-based study. Nicotine Tob Res 15, 1696–1704. 23547276 10.1093/ntr/ntt043 PMC3768332

[39] Slagter SN , van Vliet-Ostaptchouk JV , Vonk JM , et al. (2013) Associations between smoking, components of metabolic syndrome and lipoprotein particle size. BMC Med 11, 195. 24228807 10.1186/1741-7015-11-195 PMC3766075

[40] Rader DJ & Hovingh GK (2014) HDL and cardiovascular disease. Lancet 384, 618–625. 25131981 10.1016/S0140-6736(14)61217-4

[41] Yao S , Zhang R , Thornton LM , et al. (2020) Screen-detected disordered eating and related traits in a large population sample of females in mainland China: China Health and Nutrition Survey. Int J Eat Disord 53, 860–872. 10.1002/eat.23409 PMC7855662 33191528

[42] Kopin L & Lowenstein C (2017) Dyslipidemia. Ann Intern Med 167, ITC81–ITC96. 10.7326/AITC201712050 29204622

[43] Mills KT , Stefanescu A & He J (2020) The global epidemiology of hypertension. Nat Rev Nephrol 16, 223–237. 32024986 10.1038/s41581-019-0244-2 PMC7998524

[44] Culbert KM , Sisk CL & Klump KL (2021) A narrative review of sex differences in eating disorders: is there a biological basis? Clin Ther 43, 95–111. 33375999 10.1016/j.clinthera.2020.12.003 PMC7902379

[45] Gerdts E & Regitz-Zagrosek V (2019) Sex differences in cardiometabolic disorders. Nat Med 25, 1657–1666. 31700185 10.1038/s41591-019-0643-8

