# Supplementary material for: Disordered eating and cardiometabolic risk factors in Chinese women: evidence from the China Health and Nutrition Survey
Source: Br J Nutr. Author manuscript; Available in PMC 2025 Mar 23. (PMC11531933; doi:10.1017/S0007114524001983)
Supplement: 1 [file NIHMS2018116-supplement-1.docx]

**­Supplemental table 1**. Characteristics of female adults in the 2015 wave of the Chinese Health and Nutrition Survey for the full sample and those younger and older than 40 years old.

| **Demographic characteristics** | **Full sample**  **(N=2,005)** | **Age ≤ 40 years**  **(n=1,034)** | **Age > 40 years**  **(n=971)** |
| --- | --- | --- | --- |
|  | **Mean (SD)** | | |
|  |  |  |  |
| Age (year) * | 38.38 (7.97) | 32.02 (5.78) | 45.16 (2.56) |
| BMI (kg/m^2^) * | 23.51 (3.76) | 22.70 (3.80) | 24.38 (3.51) |
| Per capita household income (Chinese Yuan)† | 23,521.27 (41,480.83) | 23192.56 (32478.78) | 23875.13 (49386.56) |
| **Demographic characteristics** | **n (%)** | | |
| Geographic region * |  |  |  |
| North | 758 (37.81) | 366 (35.40) | 392 (40.37) |
| South | 1,247 (62.19) | 668 (64.60) | 579 (59.63) |
| Setting |  |  |  |
| Rural | 1,324 (66.03) | 667 (64.51) | 657 (67.66) |
| Urban | 681 (33.97) | 367 (35.49) | 314 (32.34) |
| Education†† |  |  |  |
| None or primary education * | 274 (13.67) | 76 (7.35) | 198 (20.39) |
| Middle education | 1,007 (50.22) | 521 (50.39) | 486 (50.05) |
| Technical or vocational degree * | 193 (9.63) | 119 (11.51) | 74 (7.62) |
| University degree or higher * | 405 (20.20) | 292 (28.24) | 113 (11.64) |
| **DE characteristics (cognitive)** | **Mean (SD)** | | |
| Shape concern | 0.39 (1.33) | 0.44 (1.36) | 0.35 (1.29) |
| Weight concern * | 0.27 (1.10) | 0.31 (1.17) | 0.21 (1.02) |
| **DE characteristics (behavioral)** | **Mean (SD)** | | |
| Restraint | 0.09 (0.41) | 0.10 (0.39) | 0.09 (0.42) |
| **DE characteristics (behavioral)** | **n (%)** | | |
| Loss of control eating * | 105 (5.24) | 64 (6.19) | 41 (4.22) |
| **Continuous cardiometabolic risks** | **Mean (SD)** | | |
|  |  |  |  |
| SBP (mm Hg) * | 117.26 (15.35) | 112.68 (12.44) | 122.14 (16.62) |
| DBP (mm Hg) * | 76.84 (10.10) | 74.30 (8.78) | 79.54 (10.70) |
| HbA1c (%)* | 5.40 (0.61) | 5.32 (0.55) | 5.48 (0.67) |
| Glucose (mg/dL) * | 92.75 (18.37) | 90.99 (16.63) | 94.62 (19.90) |
| Total cholesterol (mg/dL) * | 178.01 (38.18) | 170.97 (32.29) | 185.51 (37.70) |
| HDL-C (mg/dL) | 50.67 (11.58) | 50.65 (11.27) | 50.70 (11.91) |
| LDL-C (mg/dL) * | 109.28 (31.24) | 102.77 (29.06) | 116.21 (32.00) |
| Triglycerides (mg/dL) * | 98.88 (60.37) | 91.45 (59.16) | 106.78 (60.67) |
| **Binary cardiometabolic risks** | **n (%)** | | |
| Hypertension * | 258 (12.87) | 65 (6.29) | 193 (19.88) |
| T2DM * | 74 (3.69) | 26 (2.51) | 48 (4.94) |
| High total cholesterol * | 461 (22.99) | 172 (16.63) | 289 (29.76) |
| Low HDL-C | 1,012 (50.47) | 517 (50.00) | 495 (50.98) |
| High LDL-C * | 451 (22.49) | 165 (15.96) | 286 (29.45) |
| High triglycerides * | 298 (14.86) | 114 (11.03) | 184 (18.95) |

**Note:** SD: standard deviation; BMI: body mass index; DE: disordered eating; LOC: loss of control eating; SBP: systolic blood pressure; DBP: diastolic blood pressure; HDL-C: high-density lipoprotein cholesterol; LDL-C: low-density cholesterol; T2DM: type 2 diabetes mellitus.

*Variable differs significantly by age group (*p*<0.05).

†51 participants had missing data for per capita household income.

†† 126 participants had missing data for education.

**­Supplemental table 2**. Standardized regression coefficients with 95% confidence intervals from generalized estimating equations evaluating the effect of disordered eating characteristics on cardiometabolic risks, for those younger and older than 40 years old separately.

|  | **LOC** | **Restraint** | **Shape concern** | **Weight concern** |
| --- | --- | --- | --- | --- |
|  | **Age ≤ 40 years (n=1,034)** | | | |
| SBP (mm Hg) | 0.05 (-0.23, 0.32) | 0.05 (-1.53, -0.84) | 0.07 (0.00, 0.13) | 0.07 (0.01, 0.13) |
| DBP (mm Hg) | 0.23 (-0.48, 0.94) | 0.49 (-1.02, 2.00) | 0.11 (-0.15, 0.37) | 0.18 (0.02, 0.33) |
| HbA1c (%) | -0.08 (-0.30, 0.13) | 0.08 (-0.05, 0.20) | 0.06 (0.01, 0.12) | 0.09 (0.01, 0.18) |
| Glucose (mg/dL) | -0.03 (-0.21, 0.14) | 0.08 (-0.03, 0.18) | 0.05 (-0.01, 0.10) | 0.10 (0.01, 0.18) |
| Total cholesterol (mg/dL) | -0.12 (-0.34, 0.09) | 0.01 (-0.04, 0.05) | 0.02 (-0.03, 0.07) | 0.01 (-0.04, 0.06) |
| HDL-C (mg/dL) | -0.07 (-0.36, 0.22) | -0.04 (-0.10, 0.01) | -0.08 (-0.14, -0.02) | **-0.11 (-0.16, -0.05)*** |
| LDL-C (mg/dL) | -0.07 (-0.33, 0.18) | 0.04 (-0.01, 0.09) | 0.06 (0.00, 0.12) | 0.05 (-0.01, 0.10) |
| Triglycerides (mg/dL) | -0.06 (-0.31, 0.19) | 0.00 (-0.05, 0.05) | 0.04 (-0.02, 0.10) | 0.07 (0.01, 0.13) |
|  | **Age > 40 years (n=971)** | | | |
| SBP (mm Hg) | -0.12 (-0.43, 0.19) | -0.01 (-0.06, 0.04) | 0.06 (0.00, 0.13) | 0.04 (-0.03, 0.11) |
| DBP (mm Hg) | -0.01 (-0.33, 0.31) | 0.01 (-0.05, 0.07) | **0.09 (0.03, 0.15)*** | 0.07 (0.01, 0.13) |
| HbA1c (%) | 0.14 (-0.20, 0.48) | 0.05 (-0.05, 0.14) | 0.02 (-0.04, 0.09) | 0.05 (-0.03, 0.13) |
| Glucose (mg/dL) | 0.08 (-0.23, 0.39) | 0.03 (-0.06, 0.11) | 0.01 (-0.05, 0.07) | 0.04 (-0.04, 0.11) |
| Total cholesterol (mg/dL) | -0.03 (-0.34, 0.28) | -0.01 (-0.10, 0.08) | -0.02 (-0.07, 0.03) | -0.02 (-0.08, 0.05) |
| HDL-C (mg/dL) | -0.22 (-0.60, 0.16) | -0.04 (-0.12, 0.04) | -0.07 (-0.13, 0.00) | **-0.10 (-0.16, -0.04)*** |
| LDL-C (mg/dL) | 0.05 (-0.28, 0.38) | -0.05 (-0.11, 0.01) | -0.01 (-0.06, 0.04) | -0.01 (-0.07, 0.05) |
| Triglycerides (mg/dL) | 0.07 (-0.26, 0.39) | 0.05 (-0.03, 0.14) | 0.05 (-0.01, 0.11) | 0.04 (0.00, 0.09) |

**Note**: LOC: loss of control eating; SBP: systolic blood pressure; DBP: diastolic blood pressure; HDL-C: high-density lipoprotein cholesterol; LDL-C: low-density cholesterol. Models adjusted for age and education level and accounted for dependencies between individuals in the same household. **q*< 0.05.

**Supplemental table 3**. Potential standardized average total, direct, and indirect (mediating) effects with bootstrapped 95% confidence intervals in mediation models, for those younger and older than 40 years old separately.

| **Association** | **Potential standardized total effect (c)** | **Standardized a** | **Standardized b** | **Potential standardized direct effect (c’)** | **Potential standardized indirect effect (a*b)** | **(a*b)/c †** |
| --- | --- | --- | --- | --- | --- | --- |
| **Age ≤ 40 years (n=1,034)** | | | | | | |
| Weight concern – HDL-C | 0.41 (0.21, 0.61)* | 0.77 (0.55, 1.00)* | -0.07 (-0.09, -0.05) | -0.06 (-0.11, -0.01)* | -0.05 (-0.08, -0.03)* | 46% (31%, 94%)* |
| **Age > 40 years (n=971)** | | | | | | |
| Shape concern – DBP | 0.06 (0.01, 0.11)* | 0.70 (0.48, 0.92)* | 0.09 (0.07, 0.11)* | 0.02 (-0.03, 0.07) | 0.06 (0.04, 0.09)* | 74% (51%, 235%) |
| Weight concern - HDL-C | -0.45 (-0.50, -0.40)* | 0.49 (0.27, 0.71)* | -0.07 (-0.08, -0.05)* | -0.06 (-0.11, 0.00) | -0.03 (-0.06, -0.02)* | 36% (22%, 396%) |

**Note:** a = potential effect of DE on body mass index (BMI); b = potential effect of BMI on CMR; c’ = potential direct effect of DE on CMR; c = potential total effect of DE on CMR; a*b = potential indirect (mediating) effect of DE on CMR through BMI. DBP: diastolic blood pressure; HDL-C: high-density lipoprotein cholesterol; SBP: systolic blood pressure. All models were adjusted for age and education level. **p*<0.05. **†**Proportion of total effects of DE on CMR (c) accounted by indirect effects through BMI (a*b).
